# The role of Pb oxidation state of the precursor in the formation of 2D perovskite microplates†

**DOI:** 10.1039/d2nr06509f

**Published:** 2023-03-02

**Authors:** Leo Sahaya Daphne Antony, Sjoerd van Dongen, Gianluca Grimaldi, Simon Mathew, Lukas Helmbrecht, Arno van der Weijden, Juliane Borchert, Imme Schuringa, Bruno Ehrler, Willem L. Noorduin, Esther Alarcon-Llado

**Affiliations:** a AMOLF Science Park 104 1098 XG Amsterdam The Netherlands e.alarconllado@amolf.nl; b Optoelectronics Section, Cavendish Laboratory, University of Cambridge Cambridge CB2 1TN UK; c Homogeneous, Supramolecular and Bio-Inspired Catalysis, Van't Hoff Institute for Molecular Sciences, University of Amsterdam 1090 GD Amsterdam The Netherlands; d Van't Hoff Institute for Molecular Sciences, University of Amsterdam 1090 GD Amsterdam The Netherlands; e University of Freiburg, Department of Sustainable Systems Engineering – INATECH 79110 Freiburg im Breisgau Baden-Württemberg Germany; f Fraunhofer-Institut für Solare Energiesysteme ISE, Novel Solar Cell Concepts Freiburg 79110 Freiburg im Breisgau Baden-Württemberg Germany

## Abstract

Two-dimensional (2D) lead halide perovskites are an exciting class of materials currently being extensively explored for photovoltaics and other optoelectronic applications. Their ionic nature makes them ideal candidates for solution processing into both thin films and nanostructured crystals. Understanding how 2D lead halide perovskite crystals form is key towards full control over their physical properties, which may enable new physical phenomena and devices. Here, we investigate the effects of the Pb oxidation state of the initial inorganic precursor on the growth of pure-phase (*n* = 1) – Popper 2D perovskite BA_2_PbI_4_ in single-step synthesis. We examine the different crystallisation routes in exposing PbO_2_ and PbI_2_ powders to a BAI : IPA organo-halide solution, by combining *in situ* optical microscopy, UV–VIS spectroscopy and time-resolved high performance liquid chromatography. So far, works using PbO_2_ to synthesise 3D LHPs introduce a preceding step to reduce PbO_2_ into either PbO or PbI_2_. In this work, we find that BA_2_PbI_4_ is directly formed when exposing PbO_2_ to BAI : IPA without the need for an external reducing agent. We explain this phenomenon by the spontaneous reduction/oxidation of PbO_2_/BAI that occurs under iodine-rich conditions. We observe differences in the final morphology (rectangles *vs.* octagons) and nanocrystal growth rate, which we explain through the different chemistry and iodoplumbate complexes involved in each case. As such, this work spans the horizon of usable lead precursors and offers a new turning knob to control crystal growth in single-step LHP synthesis.

## Abstract 2

Lead dioxide precursor as an efficient source of highly complexed iodoplumbates for the synthesis of 2D lead halide perovskite microplates.

## Graphical abstract

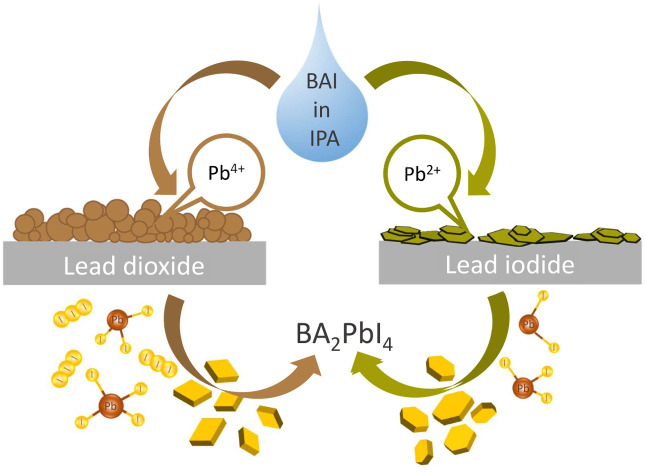

## Introduction

Low dimensional 2D lead halide perovskites (2D-LHPs) have recently attracted major attention owing to the emergence of new photo-physical properties. Compared to conventional 3D-LHPs with the chemical formula APbX_3_ (with A being an organic/inorganic cation and X a halide ion), 2D perovskites (A′_2_PbX_4_) consist of sheets of lead halide octahedra (PbX_6_)^4−^ isolated by long chain hydrophobic spacer cations (A′). As a result, 2D-LHPs are highly anisotropic and naturally form multiple quantum well super-lattice structures.^1^ Owing to the large library of organic cation spacers, 2D-LHPs offer new approaches to promote quantum confinement effects,^2,3^ large exciton binding energies,^4^ strong exciton–phonon coupling,^5–8^ giant Rashba splitting^9^ and large optical non-linearities.^10,11^ Additionally, the hydrophobic nature of the organic spacer promises 2D perovskites to be more stable in ambient conditions,^12^ which has triggered a paradigm shift in the field of perovskite photovoltaics and other optoelectronic devices.^13–18^

The ionic nature of LHPs have made their synthesis very simple using solution based techniques.^19–22^ Common solution-based synthesis involves the use of organo-halide and inorganic lead salts dissolved in a solvent (*i.e.*, precursor solution) to crystallize LHPs in the form of either thin films^22,23^ and nanocrystals.^24–28^ In 3D LHPs, it is well known that the solution chemistry and co-ordination of Pb–I complexes (iodoplumbates) in the solution play a crucial role in the crystallization and the physical properties of the resulting material (*e.g.* defect density, morphology or crystal orientation).^29^ In particular, iodine-rich and high-valency iodoplumbates (like PbI_4_^2−^, PbI_5_^3−^, …) promote higher quality 3D LHP films with lower defect density,^30^ less pinholes and larger grain sizes.^31^ As a result, better performing solar cells^32,33^ (in terms of power conversion efficiency and reproducibility)^34^ have been demonstrated by judiciously targeting solution complexation.

Various strategies have been proposed to promote the formation of highly coordinated iodoplumbate complexes, including the use of weakly coordinating solvents,^35,36^ increasing the Pb : I ratio in the solution,^37,38^ incorporating I_3_^−^ to the solution,^30^ photo-induced HI dissociation^39^ and irradiating of the precursor solution.^36^ Similarly, controlling crystallisation of 2D LHPs has been done by adjusting the solvent^40,41^ and organo-halide salt concentration,^42,43^ while the role of inorganic-lead precursors has been mostly omitted.

In this manuscript, we explore the effect of solid inorganic-lead precursors in the formation of iodoplumbate complexes and driving the crystallization of the pure-phase, *n* = 1, 2D-LHP when exposed to an organo-halide solution. Specifically, our work employs *in situ* optical imaging of BA_2_ PbI_4_ (BAPI) crystal formation and growth from two different lead precursors with different initial lead oxidation states: lead dioxide (PbO_2_), lead iodide (PbI_2_), when exposed to the same *n*-butylammonium iodide/2-propanol solution conditions. In both cases, we find heterogeneous growth of randomly-oriented BAPI flake-like crystals on the substrate and the formation of BAPI microplates in solution, with similar optical properties. However, these crystals exhibit significant qualitative differences in the growth rate and faceting depending on the Pb precursor. Furthermore, the BAPI microplates produced from PbO_2_ conversion are mono-crystalline and structurally high-quality compared to those produced from PbI_2_.

We suggest that the Pb^4+^ in the PbO_2_ precursor undergoes an spontaneous reduction to Pb^2+^ generating I_3_^−^ in solution without the need of an additional reduction step, as used in previous works.^44–46^ This in turn, promotes the formation of high-valence iodoplumbates that accelerates crystallisation. We confirm this mechanism by time-resolved high-performance chromatography and absorption spectroscopy. This work demonstrates the potential of inorganic lead sources with non-matching Pb oxidation state as *in situ* promoter of high quality 2D perovskite synthesis.

## Results

In this work we focus on the conversion to butylammonium lead iodide (BAPI) from two different Pb solid crystalline precursors: PbO_2_ and PbI_2_. The precursors PbO_2_ and PbI_2_ vary in their Pb oxidation state (+4 and +2, respectively), crystal structure (tetragonal and hexagonal) and material class (metal and semiconductor). By employing 2-propanol as the solvent, we expect the formation of high-valency iodoplumbates due to the weak co-ordination of solvent molecules with Pb^2+^ center.

**Fig. 1:**
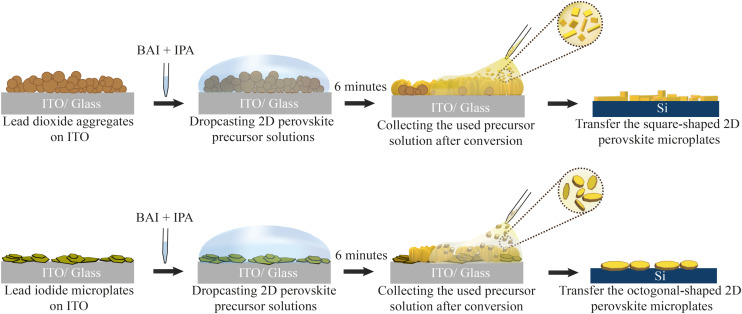
Sample fabrication process schematics based on solid lead precursors. Both lead-based aggregate films (PbO_2_, PbI_2_) on ITO were exposed to the organo-halide solution for 6 minutes. After that, the solution was collected and, if present, 2D perovskite microplates in solution are then transferred to a new silicon substrate.

Fig. 1 briefly describes the conversion process of both Pb precursors to BAPI. We start by drop-casting and sintering powder of the Pb precursors dispersed in anhydrous 2-propanol (IPA) on a clean ITO substrate. For all samples, the molarity of Pb is kept constant (see ESI section 1†). All the Pb precursor samples are non-compact aggregates of the corresponding Pb-sources (see ESI Fig. S1†) with PbO_2_ appearing black and PbI_2_ appearing yellow to the naked eye (ESI Fig. S2a†). Subsequently, we drop-cast 200 μl of organo-halide solution (0.3 M *n*-butylammonium iodide (BAI) in anhydrous IPA) on the Pb-precursor substrate in a nitrogen filled glove-box. The volume of the organo-halide solution was chosen such that it fully covers the substrate and it does not completely evaporate during the exposure (6 minutes).

During exposure, we observe the organo-halide solution undergoing a colour change from colourless to saturated yellow/brown in the case of PbO_2_ and slight yellow in the case of PbI_2_ (ESI Fig. S2b†). Additionally, as the exposure time increases, we observe speckles of light in the solution. As explained later, these indicate the presence of micrometer-sized BAPI crystals floating in solution, that reflects ambient light. After the 6 minutes of exposure, the solution is collected *via* pipette and transferred either to a filter paper or an external substrate, where the presence of small crystal-like microplates become evident to the naked eye. To characterise these floating microplates, we dry transfer them from the filter paper to any desired substrate (*e.g.*, silicon or glass) using a polydimethylsiloxane (PDMS) sheet (see ESI section 3†). At the same time, the exposed substrate is rinsed in clean IPA to remove the reaction by-products and finally dried. After the exposure, the PbO_2_ sample shows a clear change in appearance, from matte black to reflective yellow/brown indicating the possible formation of BAPI on the substrate. The PbI_2_ converted sample shows a less evident change after exposure, with dense bright yellow aggregates (ESI Fig. S2a†).

**Fig. 2:**
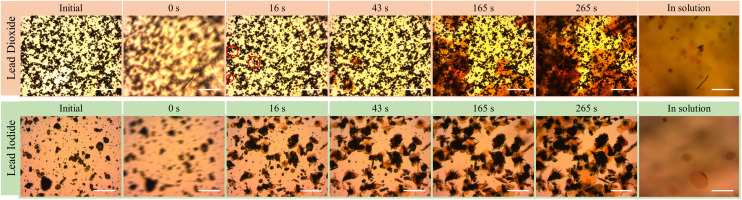
Sequence of optical microscopy images of the Pb-based samples before and during solution exposure, at the times indicated above. We consider 0 s the time at which the BAI solution is drop-casted. The right-most picture is taken by focusing on the solution above the substrate to show the presence (or not) of crystal growth in solution (scale: 100 μm). Red circles marked on row one correspond to primary nucleation sites on PbO_2_ substrate.

To gain more insights on the conversion process, we track the exposure reaction on the Pb-based samples *in situ* under the optical microscope. The microscope setup was enclosed in a portable glove-box flushed with N_2_ to ensure the reactions were performed at a controlled low humidity environment. Fig. 2 shows bright field microscopy snapshot images of the samples before and during the solution exposure. While drop-casting the organo-halide solution, we define the start of the exposure (0 seconds) once the substrate is fully covered by the solution, at which point we refocus the image on the substrate. Before conversion (left-most image), the PbO_2_ sample surface shows non-uniform dark opaque aggregates.

Within the field of view (461 × 369 μm^2^), after 16 seconds, we observe the appearance of small orange-coloured crystals on the substrate from three different nucleation points (marked with red circles), that keep growing into asymmetrical crystal clusters (see ESI Video 1†). Strikingly, we also observe the formation of square-shaped crystals in the solution (see ESI Video 2†) by lifting the focus above the substrate. These crystal sizes vary from 14–37 μm. These crystals start growing as early as 20 seconds and they exhibit a slower growth rate compared to those crystals growing directly on the substrate. The crystals in solution also retain their initial square shape as long as they are suspended in solution. From the *in situ* videos, we often see that some floating crystals adhere to the substrate, promoting secondary nucleation on the substrate (ESI Fig. S4†). As the exposure continues, these square-shaped crystals that attach to the substrate, exhibit prominent diagonal growth resulting in asymmetrical star shapes (ESI Fig. S5†).

In contrast to the PbO_2_ sample, the initial PbI_2_ sample surface consists of hexagonal-shaped dark yellow crystals and small unevenly shaped particles. During the exposure, we notice that some large PbI_2_ crystals detach from the substrate and go into the suspension. At 16 seconds, we already observe that most existing PbI_2_ crystals are surrounded by newly forming orange coloured crystals parallel to the substrate (see ESI Video 3†). Over the course of exposure, we see a steady increase in the size of these orange crystals and some out-of-plane growth of new crystal structures. In contrast to the PbO_2_ sample, the PbI_2_ sample shows a higher nucleation density of substrate-bound crystals with a slower growth rate. Similar to the previous case, we also observe the formation of crystals in solution (see ESI Video 4†), which also induce secondary nucleation after adhering to the substrate (ESI Fig. S6†). In this case, the crystals start hexagonal-shaped, continue to grow octagonal (ESI Fig. S7†) and typically grow to larger dimensions up to 140 μm.

**Fig. 3:**
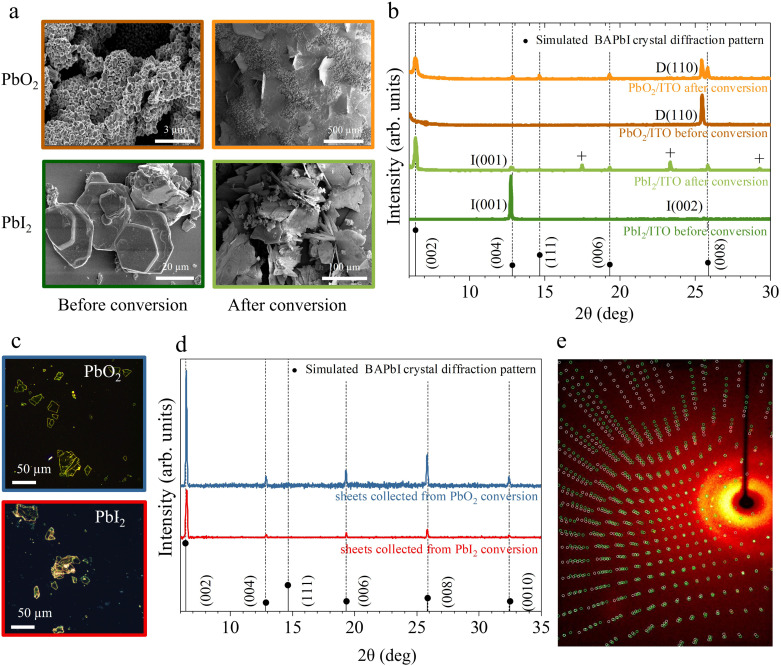
Structural and morphological characterisation. (a) SEM images and (b) powder X-ray diffractograms of the PbO_2_ (top), PbI_2_ (bottom) samples before and after exposure to BAI solution. D, I pre-factors indicate the PbO_2_, PbI_2_ phases, respectively. The “+” labeled peaks correspond to the BAI precursor. BAPI microplates isolated from the solution during conversion of PbO_2_ and PbI_2_ and transferred to glass substrates. Their corresponding (c) optical images and (d) powder X-ray diffractograms. The assigned crystal orientation for BA_2_PbI_4_ and Pb precursor phases is indicated by the labels next to each peak. (e) Single crystal diffractogram of a representative BAPI microplate collected during conversion of PbI_2_ depicting a non-merohedral twinning.

Fig. 3a shows the scanning electron microscopy images of the lead precursor substrates before and after conversion process. After conversion, both PbO_2_ and PbI_2_ substrates show the presence of large plate-like features growing both in- and out-of-plane. Additionally, PbO_2_ substrates show the presence of remaining PbO_2_ aggregates indicating that the exposure time was too short to fully convert all initial material. The crystal structure of the samples before and after the conversion reaction is confirmed using powder X-Ray diffraction (P-XRD), as shown in Fig. 3b. The BAPI crystal structure^47^ was simulated with VESTA^48^ and the expected diffraction peaks are plotted along with the measured XRD scans as a visual guide to the reflections.

The initial PbO_2_ film (dark orange curve) reveals a characteristic peak around 25.4° corresponding to the (110) plane of tetragonal β-PbO_2_ phase.^49^ The initial PbI_2_ sample (dark green curve) reveals two characteristic peaks at 12.67° and 25.47° corresponding to the (001) and (002) planes of hexagonal PbI_2_.^50^ After exposure to BAI (orange and light green curves), low angle periodic (0 0 2·*l*) reflections for *l* = 1 to 4 are visible on the XRD pattern for both substrates, indicating the partial conversion of the initial film to BAPI 2D perovskite with the *c*-plane horizontally oriented with a *d*-spacing of 1.38 nm. In addition to the (0 0 2·*l*) reflections, a peak around 14.63° is also visible, indicating the presence of vertically orientated 2D perovskite, (labeled as (111) in Fig. 3b). In both converted substrates, the P-XRD scan shows reduced intensity peaks of the original lead precursor phase which confirms that not all the lead precursors converted likely due to the limited exposure time. The PbI_2_ reflection peaks are the ones that are reduced the most, therefore suggesting a more efficient conversion into BAPI when compared with PbO_2_.

On the other hand, the BAPI microplates grown in solution during the exposure of both substrates were transferred on to clean ITO/glass substrates with a PDMS sheet. The fresh substrate was stamped multiple times with same PDMS sheet until sufficient density of crystals were transferred. This brute process usually results in cracking of the micro-plates as shown in the optical microscopy images Fig. 3c. The XRD scans of these microplates (Fig. 3d) reveal the presence of parallel oriented highly crystalline BAPI sheets with the same *d*-spacing of 1.37 nm as in the BAPI grown on the substrate.

All investigated microplates displayed uniform extinction under a polarized light microscope (*i.e.* the whole microplate switches from bright → dark → bright at the same angle of rotation between crossed polarizers). Further inspection with single crystal X-ray diffraction of a few microplates, corroborates that the BAPI microplates are monocrystalline. In all cases, a crystal structure with lattice constants consistent with the previously reported data from Mitzi *et al.*^47^ was obtained (see ESI section 6†). Analysis of the diffraction reflections as a function of angles of incidence allows for solving the full crystallographic structure. While the textbook structure was resolved in microplates grown from the PbO_2_ precursor, full scans on microplates from the PbI_2_ precursor did not yield an unequivocal crystal unit cell and we observe very low redundancy and completeness in the reflections (ESI section 7†). This indicates a superior quality of BAPI microplates grown from PbO_2_.

Fig. 3e shows a representative single crystal X-ray diffractogram of a BAPI microplate generated from PbO_2_. In this case, a small-angle twin defect was identified by the two sets of reflections shown as red and green spots in Fig. 3e, which could either indicate twin growth during the crystal synthesis or a possible secondary crystal contamination in the holder in addition to the crystal under observation.

**Fig. 4:**
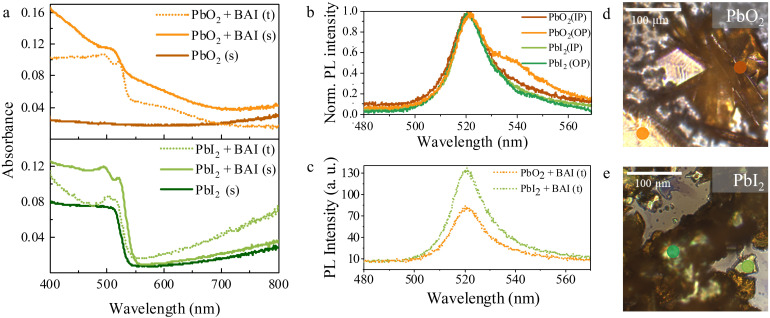
Optical characterisation of the samples before and after conversion. (a) UV–Vis absorption spectra of the lead precursor samples before and after conversion (solid curves) along with those for the solution-grown crystals transferred on ITO (dashed curves). Photoluminescence spectra of the (b) converted substrates and (c) crystals grown in solution after transfer on ITO, respectively. IP, and OP represent crystals grown in-plane and out-of-plane on the substrate respectively whereas s and t denote crystals formed on the substrate and transferred crystals from solution, respectively. Optical images and laser spots with representative color code of the converted (d) PbO_2_ and (e) PbI_2_ substrates showing IP, and OP crystals.

We now focus on the optical properties of the samples before and after exposure, as well as for the transferred microplates formed in solution. Fig. 4a shows the absorbance spectra from 400 to 800 nm for the PbO_2_ and PbI_2_ precursor samples before conversion in dark-orange and dark-green respectively, and after conversion in light-orange, light-green. The absorption in the initial PbO_2_ precursor substrate is low and fairly constant over the whole spectrum, except for a steady rise in the long wavelengths due to free-carrier absorption in the ITO layer. In contrast, the PbI_2_ precursor sample shows an absorption edge around 530 nm (2.34 eV), in line with the reported bandgap of thin PbI_2_ crystals (2.3–2.5 eV).^51^

After exposure, all converted substrates (solid lines) and transferred microplates (dashed lines) show similar room temperature absorption spectra with a continuum of absorption at short-wavelengths and a pronounced primary excitonic peak at 522 nm, which is very close to values previously reported in literature for BAPI.^3,5,7^ Consistent with previous research,^3,52^ an additional exciton-like peak is observed at 495 nm (2.5 eV). In some cases, this absorption feature is so broad that it overlaps with the primary exciton peak. In the PbO_2_ case, a broad absorption feature around 550–650 nm also appears in both the converted substrate and solution-grown sheets, which has previously been attributed to either energetic disorder^5^ or to the presence of amorphous PbI_2_.^53^ This observation suggests that the growth from PbO_2_ involves an amorphous PbI_2_ intermediate or that the final BAPI is more susceptible to degradation.

Light emission from the converted substrates and the transferred microplates is further investigated by steady state photoluminescence (PL) microscopy, as shown in Fig. 4(b) and (c), respectively. Under 405 nm excitation, all samples show green PL emission. In the substrate converted samples from both precursors (Fig. 4b), we observe the contribution of two peaks to the PL signal, where the most intense is centered at 521 nm and it is consistent with the excitonic peak in absorption spectra. Additionally, a distinct low-energy side-band centered around 540 nm is also observed, which is particularly prominent in the dark green and orange curves in Fig. 4b. Recent work has shown that radiation from out-of-plane bright magnetic dipole transitions in BAPI at this energy is enabled by off-normal incidence excitation.^54,55^ This origin is consistent with the fact that the low-energy band PL intensity in both substrates varies depending on whether horizontally or vertically oriented BAPI sheets are probed as shown in the optical images of substrates (see Fig. 4d and e).

Representative PL emission from individual microplates grown in solution (Fig. 4c) shows a single asymmetric peak centered at 521 nm. Such asymmetric PL emission in BAPI has been previously observed,^5,26^ with the asymmetry being suppressed at low temperatures due to strong exciton–phonon coupling.^5^ The absence of the low-energy band in the solution-grown crystals is consistent with the fact that the transferred sheets lay horizontally on the host substrate. The similar emissions from microplates grown in solution highlights the similar quality of both BAPI microplates irrespective of their initial lead precursor.

## Discussion

To summarize, we have shown that crystalline BAPI is formed when simply exposing either of the lead precursors to BAI : IPA solution. Yet, we observe clear differences in the BAPI crystallization from PbO_2_ and PbI_2_:

(i) Growth rate of BAPI crystals; based on the *in situ* observations, BAPI crystal growth on the substrate is faster in PbO_2_ than PbI_2_.

(ii) Colour change of the dropcasted BAI solution; solution exposing PbO_2_ experiences a stronger color change from clear to yellowish brown, whereas the PbI_2_-exposed solution only turns slightly yellow.

(iii) Morphology of solution-grown BAPI crystals; even though BAPI crystals on both the PbO_2_ and PbI_2_ substrates occurs in the form of sheet-like crystals, solution-grown BAPI crystals form in the shape of either a square or hexagon for the PbO_2_ and PbI_2_ cases, respectively.

All the differences listed above are likely due to different crystallization pathways induced by the different chemistry of the precursors. Previous works^56^ have demonstrated that the layered nature of the PbI_2_ crystal structure makes it easy for the intercalation of the organic molecule and subsequent topotactic growth into the perovskite form,^57^ following a reaction such as:

1 PbI_2_(s) + 2[C_4_H_9_NH_3_I](soln) → (C_4_H_9_NH_3_)_2_PbI_4_(s)

**Fig. 5:**
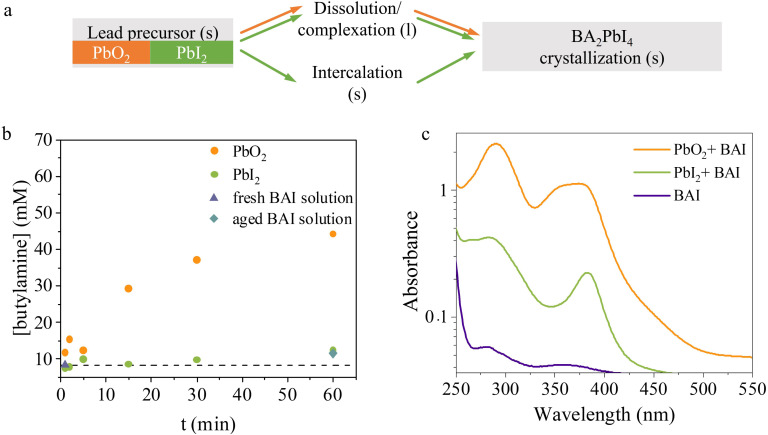
(a) Proposed crystallization pathways of the different lead precursors in solution-mediated conversion to BAPI. s and l refer to the solid and solvated form, respectively. The main crystallisation pathways for each of the lead precursors are indicated by colors; orange for PbO_2_ and, green for PbI_2_. (b) Time-dependent HPLC-UV detection of butylamine generated in solution during the conversion reaction of PbO_2_ and PbI_2_ with BAI. (c) Absorption spectroscopy of solutions collected/filtered during the conversion reaction of PbO_2_ and PbI_2_ with BAI.

A similar reaction pathway is of course not possible in PbO_2_ given its very different crystal symmetry and Pb oxidation state compared to BAPI. On the other hand, PbI_2_ is highly soluble in excess of iodide ions.^56,58^ Given that we also observe BAPI crystallisation in the solution itself and we cannot rule out a topotactic reaction in our PbI_2_ films, we suggest that the PbI_2_ precursor when exposed to the solution undergoes a kinetic competition between intercalation of BA-ions from the solution and dissolution/re-crystallisation. These crystallisation paths are summarised in Fig. 5a.

The ability of a precursor to go into solution is ruled by its solubility in the given solvent. Although both Pb precursors used here are insoluble and tend to form a suspension in IPA, it has been shown that the presence of excess iodide ions in IPA facilitates the solvation of PbO_2_ and PbI_2_ precursors to form Pb–I coordinated complexes.^56,59^ In these works, excess iodine was introduced in the form of HI, and the dissolution rate was shown to be strongly influenced by the Pb : I ratio, where the more I, the more the dissolution. In our case, excess iodine only arises from the BAI molecule dissociation into BA^+^·I^−^. In all experiments we have kept the same molar ratio of Pb and BAI at 1 : 14. BAI solution dissociation is verified through UV–VIS absorption measurements of a fresh BAI : IPA solution, shown by the purple curve in Fig. 5c. The spectrum shows two small peaks centered around 290 nm and 360 nm that have been previously assigned to the presence of I_3_^−^ ions in IPA.^30^

The formation of I_3_^−^ ions could be attributed to the light induced BAI degradation, similar to what was previously seen in 3D perovskite MAI precursor solutions.^39^

Previous works have shown the formation of 2D and 3D perovskites in solution from PbI_2_ thin-films through dissolution, complexation and precipitation reactions as follows:^56^

2 PbI_2_(s) + I^−^(soln) ⇌ PbI_3_^−^(soln)

3 PbI_3_^−^(soln) + I^−^(soln) + 2[C_4_H_9_NH_3_]^+^(soln) → (C_4_H_9_NH_3_)_2_PbI_4_(s)

A similar reaction pathway is not possible with PbO_2_ due to the Pb^4+^ oxidation state. Based on our observations and existing literature on dissolution of Pb-precursors^60^ we suggest the following chemical reaction pathway during the BAI : IPA exposure of PbO_2_. First, the PbO_2_ precursor undergoes a spontaneous primary redox step with I^−^ that yields Pb^2+^, triiodide (I_3_^−^), butylamine (C_4_H_9_NH_2_) and water into the solution as follows:4
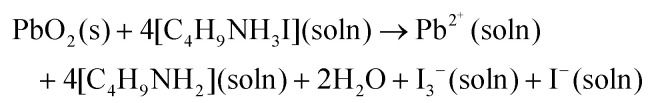
which is then succeeded by the complexation of Pb^2+^ ions into high-valent iodoplumate species *via*

5 Pb^2+^ + 3I^−^ ⇌ PbI_3_^−^

or

6 Pb^2+^ + 4I^−^ ⇌ PbI_4_^2−^

Subsequently, BAPI crystals precipitate through the reaction of iodoplumbate species with BA^+^ following either eqn (3) or:

7 PbI_4_^2−^(soln) + 2[C_4_H_9_NH_3_]^+^(soln) → (C_4_H_9_NH_3_)_2_PbI_4_(s)

It is interesting to note that compared to the PbI_2_ precursor case, there is now additional BA, I_3_^−^ and water in solution. While small water concentrations may help promote BAI dissociation, and therefore increase the concentration of I^−^ species, too much water would be detrimental for BAPI precipitates. The role of water in the reaction is further elaborated in the ESI section 10.†

On the other hand, Yang *et al.*^30^ observed that the presence of added I_3_^−^ during FAPbI_3_ formation improved device performance through the elimination of deep level defects. More recently, other works suggested that introducing I_3_^−^ during 3D perovskite formation helps prevent/regenerate point defects, like metallic lead.^61,62^ This indicates that the spontaneous iodine oxidation when using PbO_2_ as precursor naturally provides the right chemical environment to produce high quality BAPI.

To validate eqn (4), we track the presence or absence of the butylamine reaction side product (C_4_H_9_NH_2_ or BA), as BA is released only during PbO_2_ reduction (according to eqn (4)). High performance liquid chromatography coupled with a UV-detector (HPLC-UV) was used to identify the BA in the BAI solution, which is periodically collected over the course of the reaction. More details on the experiment and detection process is described in the ESI.† The amount of BA found in a fresh and aged BAI solutions (shown as purple triangles and blue rectangles, respectively in Fig. 5b) is used as the baseline of the BA arising from dissociation of *n*-butylammonium salt dissolved in IPA. In both cases, the signal is within the noise level and it indicates a minimal or no dissociation of the salt in IPA into BA consistent with similar reports.^63^

The BA measured in aliquots of reaction solution obtained at various time intervals after PbO_2_ and PbI_2_ precursors are exposed to fresh BAI solution are shown as the orange and green dots, respectively in the Fig. 5b. As expected, the PbI_2_ precursor doesn't release any detectable amount of BA in the solution over the course of the sampling interval. Yet, when the collected aliquots of the reaction solution were inspected after a day, we found the presence BAPI crystals in them (Fig. S14†). This further confirms that the synthesis of BAPI crystals in solution from PbI_2_ precursors is *via* non-butylamine mediated reaction scheme as proposed in reactions (2) and (3), where iodoplumbate species are released into solution. On the other hand, the solution with PbO_2_ precursor is shown to release BA into the solution, which steadily increases with time over the probing period. This confirms that our proposed redox reaction (4) takes place in the solution, and again releasing lead ions into solution with the right oxidation state.

As mentioned earlier, the organo-halide solution experienced a dynamic colour change (also shown in ESI Fig. S2†) during exposure of the PbO_2_ and PbI_2_ samples. It has been extensively reported that Pb–I coordinated complexes have specific absorption bands in the visible spectral range. The central wavelength of these bands depends on the level of Pb–I coordination, spanning from yellow to dark brown as the valence state increases.^35,37,64,65^

In our case, PbO_2_ leads to the darkest solution in comparison with PbI_2_, indicating the rapid release of highly coordinated Pb–I complexes, as indicated by eqn (5) and (6). The absorption spectra of this solution (orange curve in Fig. 5c), shows multiple absorption maxima at 290 and 350 nm, indicating the high presence of I_3_^−^ ions and at 380 and 450 nm, related to PbI_3_^−^ and PbI_4_^2−^ iodoplumbate species, respectively. Such highly coordinated iodoplumbate species can be strongly reactive, therefore leading to the fast precipitation of crystals in solution.

By contrast, the absorption spectra of the reaction solution collected when PbI_2_ precursor reacts with BAI solution (green curve in Fig. 5c) does not show I_3_^−^-related features (*i.e.* peak at 350 nm) and the absorption is negligible for wavelengths longer than 450 nm. The latter indicates the lack of iodoplumbate species with coordination of PbI_4_^2−^ and higher. The absorption spectra thus further corroborate the different BAPI formation mechanisms in PbO_2_ and PbI_2_.

Finally, the fact that the shape of solution-grown crystals is different in the cases of PbI_2_ and PbO_2_ precursors also can be an indirect consequence of difference in the crystal system of the precursors. Apart from homogeneous nucleation in solution, BAPI nucleation centers may also start forming at the droplet/air interface given the reduced nucleation barrier^66^ in both precursor substrates.

## Conclusions

In summary, we have shown that 2D perovskite BAPI crystals can be rapidly and easily formed at room temperature from various solid Pb precursors, irrespective of their Pb oxidation state. The PbO_2_ conversion process can be exploited as a simple synthesis alternative to complex exfoliation processes for making high-quality monocrystalline BAPI sheets. We observe that there is a competition between the chemical conversion of the host Pb-based solids and dissolution/re-crystallization reactions that defines the chemical pathway towards the final BAPI. Based on HPLC-UV and the optical characterisation, we argue that the solubility of the lead precursors in halide-rich solutions is a key parameter that rules solution-based processing of 2D-LHPs. This work also highlights the use of PbO_2_ lead precursor as an *in situ* source of high valency iodoplumbate complexation favouring crystallization of BAPI and I_3_^−^ ions which help improve material quality. Future work will be needed to ascertain whether the conversion of PbO_2_ films can be optimised to yield high quality 2D-LHP thin films with potential applications in 2D-LHP in PV applications.

## Experimental

*X-Ray diffraction (XRD)*: XRD patterns of the converted substrates and the microplates were recorded using X-ray diffractometer, Bruker D2 phaser, with Cu Kα (*λ* = 1.54148 Å) as the X-ray source, with 0.025° (2*θ*) as the step size. *Optical microscopy*: Optical bright-field images were obtained using Zeiss, AxioCam ICc 5 optical microscope equipped with a 10.20×/0.2 objective EC Epiplan. *Scanning electron microscopy (SEM)*: Scanning electron microscopy images were captured with an FEI Verios 460 at 5 kV, 500 pA e-beam current. *UV*/*visible absorption spectroscopy (UV-Vis)*: UV-Vis spectra was measured using integrated sphere and a LAMBDA 750 UV/Vis/NIR (near-infrared) spectrophotometer (PerkinElmer). The sample was placed with an angle of 18° to obtain the fraction of absorbed light. The solution absorbance measurements where performed by use of custom-fit quartz flow cell to hold small measurement volume. *Microscopic photoluminescence spectroscopy (PL)*: PL spectra/spatial maps was obtained using a WITec alpha300 SR in combination with a Thorlabs S1FC405 405 nm laser as the excitation source. A 488 nm long pass filter was used to remove the excitation laser from the signal. The reflected light was collected using a 0.9 NA objective from the same side of sample excitation and coupled to a UHTC 300 VIS (WITec) spectrometer. The laser power was always keep at 0.10 mW unless stated otherwise. *High performance chromatography with UV detector (HPLC-UV)*: HPLC-UV analysis was performed using an Agilent Technologies Infinity 1260 HPLC system equipped with a Chiralpak IA (250 × 4.6 mm, 5 μm) column, eluent: heptane/isopropanol 70/30 (v/v), flow rate: 0.7 mL min^−1^ (UV detection: 220 nm). *Single crystal X-ray diffraction*: X-ray diffraction data of BAPI microplates were measured on a Bruker D8 Quest Eco diffractometer using graphite-monochromated (Triumph) Mo Kα radiation (*λ* = 0.71073 Å) and a CPAD photon III C14 detector. The sample was cooled with N_2_ to 100 K with a Cryostream 700 (Oxford Cryosystems). Intensity data were integrated using the SAINT software. Absorption correction and scaling was executed with SADABS. The structures were solved using intrinsic phasing with the program SHELXT 2018/2 against *F*^2^ of all reflections. Least-squares refinement was performed with SHELXL-2018/3. All non-hydrogen atoms were refined with anisotropic displacement parameters. The hydrogen atoms were introduced at calculated positions with a riding model.

## Author contributions

A. v. d. W. did the substrate preparations. L. S. D. A. carried out the *in situ* optical imaging, measurements and processed the experimental data. S. M. performed the single crystal XRD measurements and data processing. S. v. D. performed the HPLC-UV measurements. E. A. L., W. L. N., B. E. were involved in planning and supervision of the work. J. B. and I. S. helped with writing – review. L. S. D. A., G. G., L. H. initiated the project. All authors contributed to the data interpretation and writing of the manuscript.

## Conflicts of interest

There are no conflicts to declare.

## Supplementary Material

NR-015-D2NR06509F-s001

NR-015-D2NR06509F-s002

NR-015-D2NR06509F-s003
