# Supplementary material for: The role of Pb oxidation state of the precursor in the formation of 2D perovskite microplates
Source: Nanoscale. 2023 Mar 2;15(13):6285–94. doi: 10.1039/d2nr06509f (PMC10065060; doi:10.1039/d2nr06509f)
Supplement: NR-015-D2NR06509F-s001 [file NR-015-D2NR06509F-s001.pdf]

Electronic Supplementary Material (ESI) for Nanoscale.
This journal is © The Royal Society of Chemistry 2023

Supplementary Information: The role of Pb oxidation state of the precursor in the formation of 2D perovskite microplates

Leo Sahaya Daphne Antony[1], Sjoerd van Dongen[1], Gianluca Grimaldi[1,2], Simon Mathew[3], Lukas Helmbrecht[1], Arno van der Weijden[1], Juliane Borchert[5,6], Imme Schuringa[1], Bruno Erhler[1], Willem L. Noorduin[1, 4], Esther Alarcon Llado[1,*]

1. Center for Nanophotonics, AMOLF, Science Park 104, 1098 XG Amsterdam, The Netherlands
2. Optoelectronics section, Cavendish Laboratory, University of Cambridge, Cambridge, CB2 1TN, United Kingdom
3. Homogeneous, Supramolecular and Bio-Inspired Catalysis, Van't Hoff Institute for Molecular Sciences, University of Amsterdam, 1090 GD Amsterdam, The Netherlands
4. Van't Hoff Institute for Molecular Sciences, University of Amsterdam, 1090 GD Amsterdam, The Netherlands
5. University of Freiburg, Department of Sustainable Systems Engineering - INATECH, Baden-Württemberg, 79110 Freiburg im Breisgau, Germany
6. Fraunhofer-Institut für Solare Energiesysteme ISE, Novel Solar Cell Concepts Freiburg, Baden-Württemberg, 79110 Freiburg im Breisgau, Germany

1 Fabrication of the lead precursor/ITO substrates

Lead(IV) oxide (CAS:1309-60-0) and Lead(II) iodide (CAS:10101-63-0) was purchased from Sigma-Aldrich. To make different lead precursor films on ITO, corresponding 22mM of lead precursor suspensions were made in anhydrous isopropanol. These precursor suspensions were sonicated to form a stable dispersion. ITO substrates ($1.5 * 1.5$ cm^2) were multi-step cleaned with soap water, acetone, isopropanol, deionized water and dried with a nitrogen gun. Cleaned ITO substrates were placed on a hotplate at 58 degrees and the lead precursor solutions were dropcasted on the substrates. After the solvent evaporated (10 minutes) the substrates were removed from the hotplate. The size of the resulting PbO_2 NPs is estimated to be approximately 200 to 300 nm. The PbI_2 nano-plates are roughly 13-17 nm thick and measure 10-50 μm laterally.

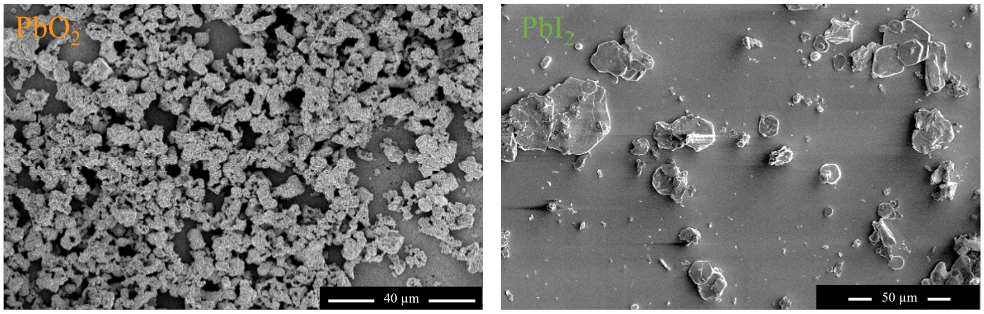

Fig. S1 SEM images of lead precursor films dropcasted and sintered on ITO (a) PbO_2 particle cluster, (b) PbI_2 hexagonal plates

2 Preparation of organo-halide solutions

n-butylammonium iodide (BAI, CAS Number:36945-08-1) was purchased from Great solar materials.The 0.3M of organo-halide solution was prepared by dissolving 0.302g of BAI in 5ml of anhydrous 2-propanol in a nitrogen filled glove-box. The solution was stirred for 10 minutes at room temperature.

3 Transfer of the microplates after the conversion

The conversion process is initiated in the nitrogen filled glove-box by drop casting the organo-halide (BAI) solution on the Pb-precursor substrate. The dissolution and re-crystallization of the PbO_2 and PbI_2 films lead to the formation of pure-phase 2D perovskite (BAPI) in the leftover precursor solution. To recover the microplates, the used precursor solution from both substrates were collected from the substrate by a clean pipette and dropcasted on a clean filter paper (Whatman filter

paper, Grade 2 or KimTech Laboratory wipes) to remove the traces of 2D precursor solution, as shown in figure Figure S2 (b). After the filter paper looks dry, the microplates are picked up from the paper using a clean Polydimethylsiloxane (PDMS) piece.

The PDMS containing the microplates can be then used to transfer the microplates onto new substrates (like Silicon, glass) as shown in figure S3 (a). If the used precursor solution with microplates are dropcasted on a clean substrate without intermediate filtering step, the traces can be visible on both optical images and XRD as shown in figure S3 (b, c). However the microplates obtained by the direct drop cast method are much larger in spatial dimensions when compared to the filter/transfer step yield.

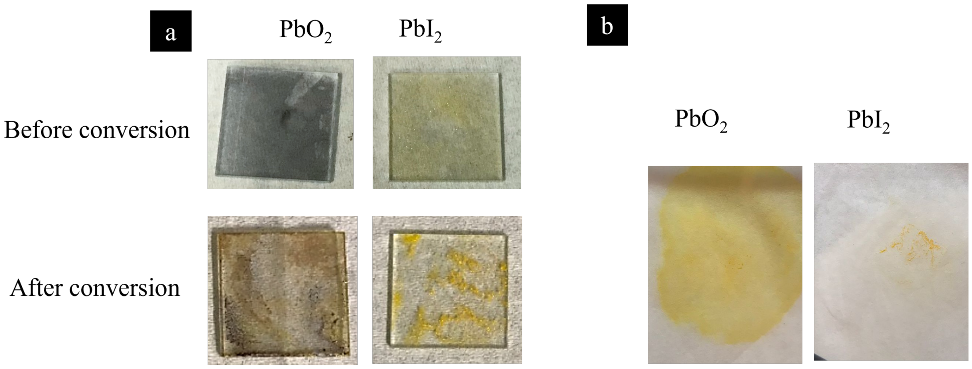

Fig. S2 Pictures of different lead precursor samples (a) before and after conversion, (b) Residues of used precursor solution after the conversion reaction on the corresponding substrate: shows the colour differences after the reaction and presence of crystals formed in the solution case of dioxide and iodide substrates

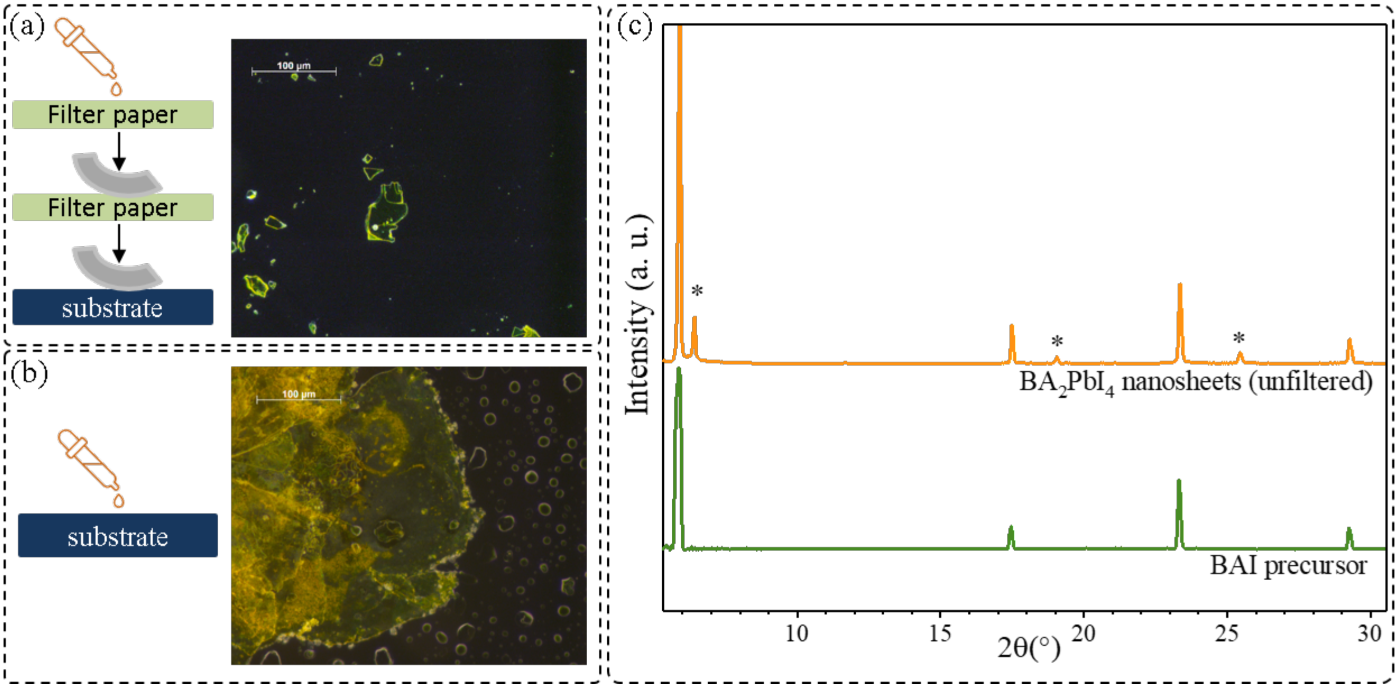

Fig. S3 Transfer of BA_2PbI_4 microplates (a) using a intermediate filtration step and subsequent dry transfer of the microplates from the filter paper to clean a glass substrate with a PDMS microplate, optical image after transfer, (b)by direct drop-casting of the collected precursor solutions on a glass substrate, optical image shows a lot of precursor residue droplets and (c) XRD spectra of the BAI precursor dropcasted on glass substrate and the BA_2PbI_4 transferred on a glass substrate without filtration step (Note: The 2D peaks are indicated by *)

4 Secondary nucleation and diagonal growth during the conversion of lead dioxide substrate

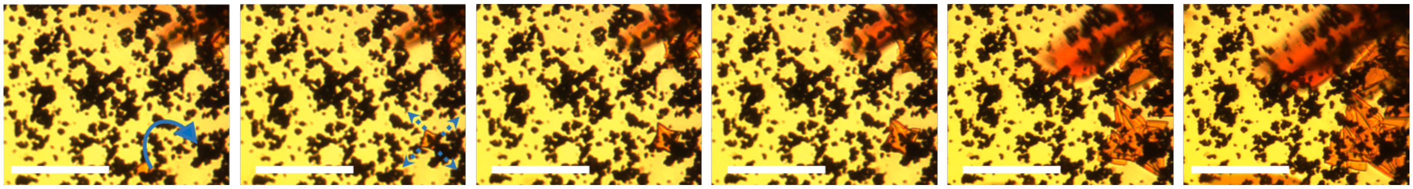

Fig. S4 Secondary nucleation initiated by the crystals formed in solution after trapped on the PbO_2 aggregates and subsequent growth (shown by blue dashed arrows) on lead dioxide substrate (Scale: 100 μm).

The secondary nucleation in the case of PbO_2 is typically promoted by the square BAPI crystals that form in solution which eventually drop to the substrate and start growing quickly along the diagonals similar to the primary nucleation that also exhibit diagonal oriented growth as seen in S5.

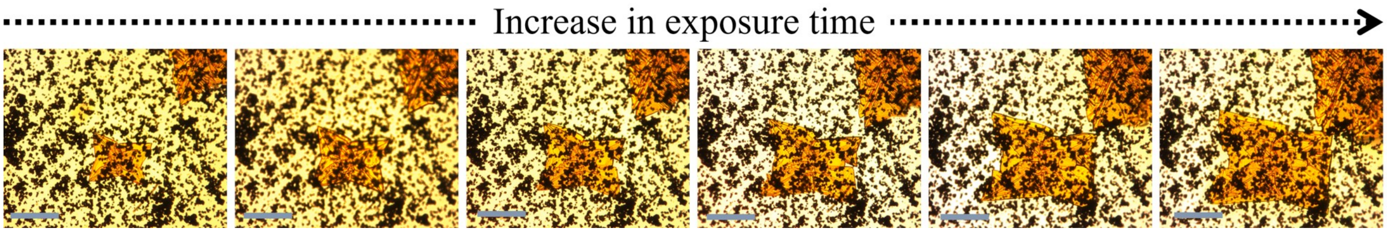

Fig. S5 Uneven diagonal growth of primary nucleation areas on lead dioxide substrate(Scale: 100 μm).

5 Secondary nucleation and hexagonal crystals seen during the conversion of lead iodide substrate

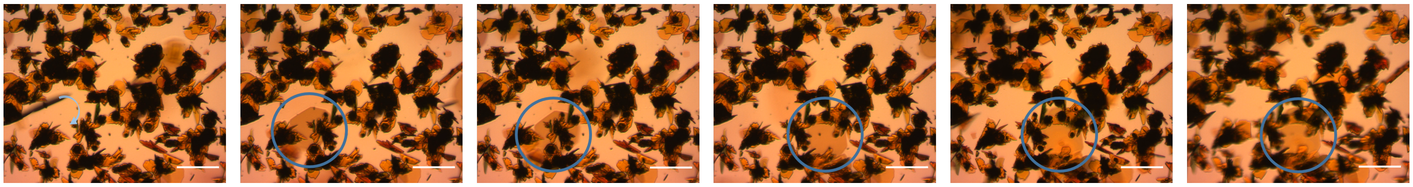

Fig. S6 Secondary nucleation during conversion of PbI_2 substrate, initiated by the crystals that originally form in solution (Scale: 100 μm). Blue curve shows the initial area where crystal formed in the solution dropped on the substrate. Blue circles shows the movement and growth of the same crystal over time (Scale: 100 μm).

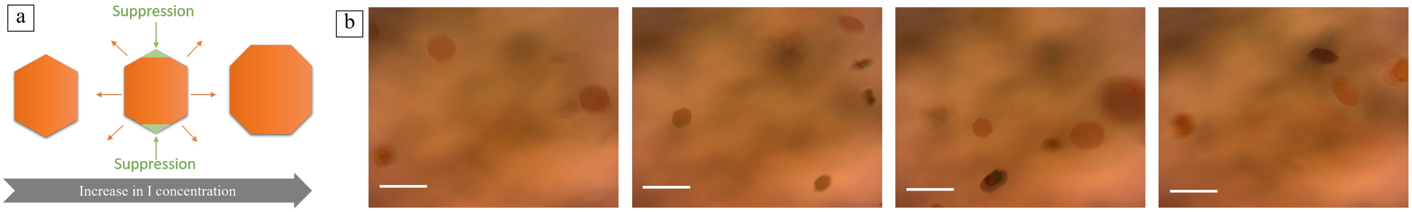

Fig. S7 Growth of crystals in solution during conversion of PbI_2 substrate, (a) schematics explaining the transformation of hexagonal shaped crystals into octagonal shaped crystals over time, with the increase in iodide concentration in the solution, (b) snapshots of droplet solution during the conversion showing various sizes of hexagonal shaped crystals (Scale: 100 μm).

6 Single crystal XRD on BAPI microplate synthesised from PbO_2 conversion

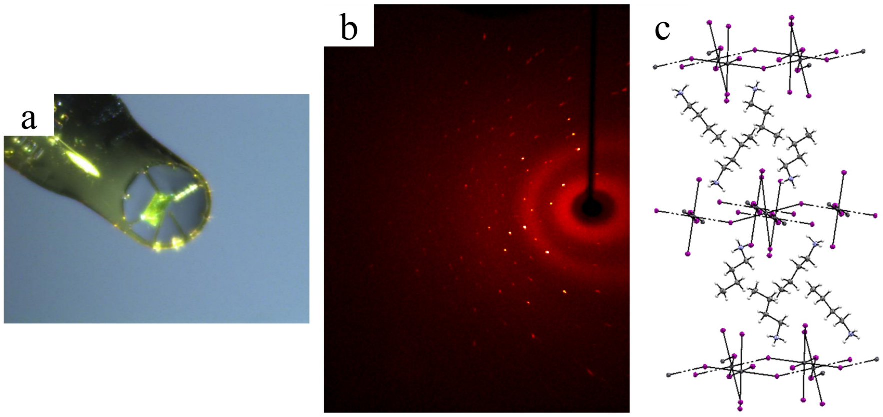

d

Crystal Size = 0.021 × 0.116 × 0.245 mm

Unit Cell Dimensions
a = 8.4191(4)
b = 8.9547(4)
c = 26.1508(12)
a = 90
b = 90
g = 90

Unit Cell Volume = 1971.52(16) A^3
Space Group Crystal System = Pbca (Orthorhombic P)

Fig. S8 (a) BAPI microplates synthesised from PbO_2 conversion used for the Single crystal X-ray diffractography, its corresponding (b) single crystal X-ray diffractogram, (c)3D unit cell structure and (d) unit cell parameters.

7 Single crystal XRD on different BAPI microplates synthesised from PbI_2 conversion

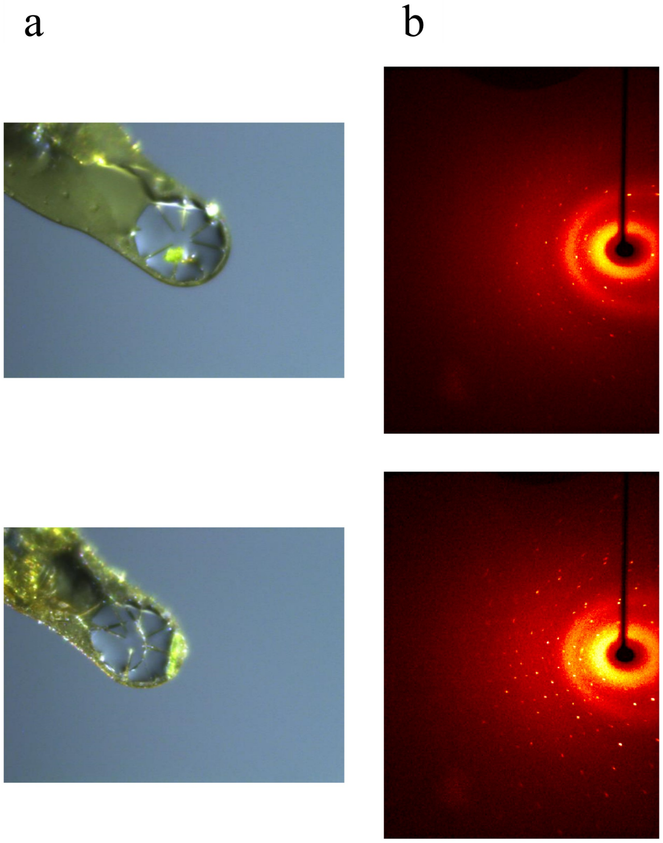

c

Crystal Size = 0.022 × 0.118 × 0.142 mm

Unit Cell Dimensions
a = 8.4167(8)
b = 8.9790(9)
c = 26.177(3)
a = 90
b = 90
g = 90

Unit Cell Volume = 1978.3(3) A^3
Space Group Crystal System = Orthorhombic P

Crystal Size = 0.042 × 0.093 × 0.320 mm

Unit Cell Dimensions
a = 8.957(8)
b = 8.400(7)
c = 26.01(3)
a = 90
b = 90
g = 90

Unit Cell Volume = 1956(3) A^3
Space Group Crystal System = Orthorhombic P

Fig. S9 (a)Two different BAPI microplates synthesised from PbI_2 conversion used for the Single crystal X-ray diffractography, their corresponding (b) single crystal X-ray diffractograms and (c) unit cell parameters.

8 High-performance Liquid Chromatography (HPLC) characterization of conversion kinetics

8.1 HPLC Analysis Protocol

HPLC analyses were performed on an Agilent Infinity 1260 HPLC with a Chiralpak IA (250 x 4.6 mm, 5 um) column. Eluent was heptane:IPA (70:30) with a flowrate of 0.7 mL/min. Detection at 220 nm (UV detector). Analyses were run for 12 minutes. Retention times were as follows: 4.8 min (the resulting imine), 5.8 min (benzaldehyde reagent), 8.2 min (R-BCA internal standard). Note: Butylamine has no UV absorption and is thus not directly detected by HPLC-UV. The compound R-BCA is a non-reacting internal standard, fully named (R)-2-(benzylideneamino)-2-(2-chlorophenyl)acetamide, that has a crucially non-overlapping retention time and allows for proper quantitation of imine concentrations.

8.2 Derivatization Procedure

The derivatization reaction of a sample containing butylamine to the UV-detectable imine by reaction with benzaldehyde proceeds as follows. A 2 mL HPLC vial is filled with 900 μL isopropanol and then 20 μL of the sample is added. Finally, 80 μL of benzaldehyde solution (500 mM in isopropanol, also containing 1 mg/mL R-BCA as an internal standard) is added and the vial is vortexed thoroughly. After 120 minutes, the vial is submitted to the HPLC for analysis. Reference chromatograms showing the different elution times of the resulting imine and the unreacted benzaldehyde reagent are shown in figure S10 (based on a derivatization of a stock solution of butylamine (10 mmol/g in isopropanol)).

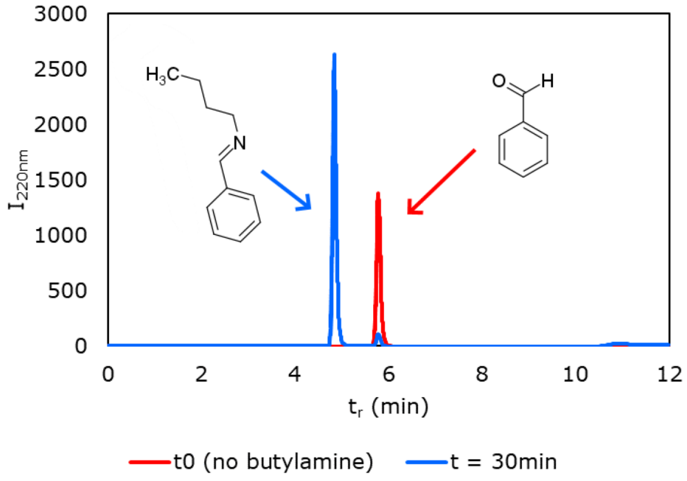

Fig. S10 Chromatograms of the benzaldehyde reagent (t0, no butylamine present) and the formed imine (product of the derivatization procedure). Detection occurs at 220 nm (HPLC UV-detector).

8.3 Calibration experiment and butylamine concentration determination

In order to calculate the concentration of butylamine in a sample, a calibration curve was constructed. To this end, stock solutions of butylamine in isopropanol were made with concentrations between 0 and 300 mM. The stock solutions were then analyzed as described under 'Derivatization Procedure' and submitted to the HPLC (injection after exactly 120 minutes). The resulting calibration curve is shown in figure S11. The ratio between the imine peak area and the internal standard peak area (R-BCA) is taken as the response (internal standard procedure); example chromatogram shown as figure S12. The calibration is proper (R2 = 0.995) and abides by the relationship,

$$[\text{butylamine}](\text{mM}) = 45.9 \cdot (\text{Peak area of imine})/(\text{eak area of R-BCA}) \qquad (8)$$

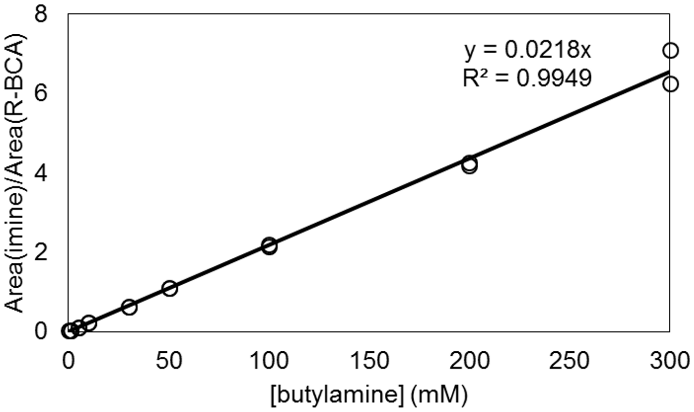

Fig. S11 Calibration curve for the determination of butylamine concentration by using HPLC, based on R-BCA as an internal standard and the detection of a derivatized imine species.

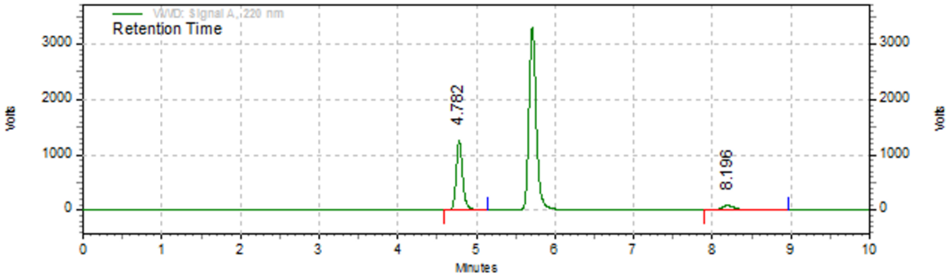

VWD: Signal A, 220 nm Results

Retention Time	Area	Area %	Height	Height %
4.782	119074750	87.63	20989801	93.81
8.196	16804251	12.37	1384022	6.19
Totals	135879001	100.00	22373823	100.00

Fig. S12 Representative chromatogram after derivatization (sample: 300 mM butylamine), showing the integration of the imine (t = 4.58 to 5.15 min) and internals standard (t = 7.88 to 8.98 min) peaks.

8.4 Derivatization kinetics validation

To validate that the derivatization reaction is completed within 120 minutes after combining the derivatization reagents, the response (ratio between imine and internal standard peak area) was measured as a function of derivatization time. This experiment was performed by injecting the product resulting from the 'Derivatization Procedure' for a 300 mM butylamine reference sample at several points in time. The results of this experiment are shown in figure S13. Fitting the data with standard chemical reaction kinetics equations (conversion 1˘$exp(-k \cdot t)$) allows for estimating that completion of the derivatization is beyond 90% after 120 minutes. For samples with lower concentrations of butylamine than 300 mM, the degree of completion is known, from theory, to increase vastly.

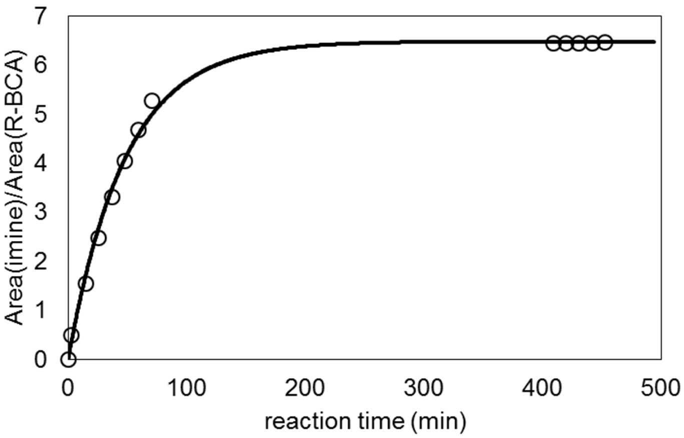

Fig. S13 Derivatization reaction kinetics for a sample of 300 mM butylamine in IPA. Conversion after 120 minutes is beyond 90% completion. Note: Completion is inversely proportional to [butylamine].

8.5 Conversion kinetics for perovskite formation

The conversion reaction is performed in the glovebox. In a 7 mL vial, 10.52 mg of PbO_2 or 20.28 mg of PbI_2 powder was weighed and 2 mL of 300 mM butylammonium iodide solution in IPA was added. At various points in time (1, 2, 5, 15, 30, 60 minutes), a 200 μL sample of the slurry was taken and filtered using a 0.2 μm PTFE-syringe filter. The filtrate was stored in a 2 mL HPLC vial and transported outside the glovebox. This sample was then immediately taken for butylamine concentration analysis using the procedure stated under 'Derivatization Procedure'.

It was earlier reported that subjecting a butylammonium iodide solution to the derivatization procedure does not lead to significant imine formation.[1] This statement was confirmed in this study as well, by subjecting the butylamine solution to the derivatization procedure. So, as a control, samples of the initial butylammonium iodide solution and of the aged butylammonium iodide solution were also subjected to analysis to provide a baseline correction.

9 Aliquots of PbI_2 reaction with BAI solution collected at different time intervals

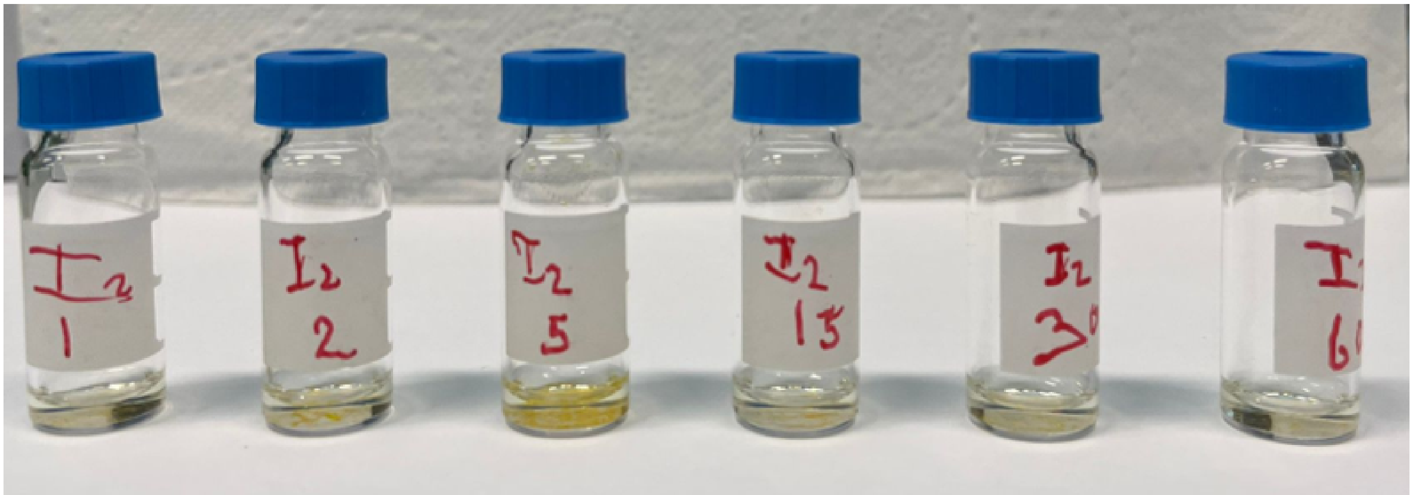

Fig. S14 Reaction solution of PbI_2 precursor and BAI solution, collected and filtered at 6 six different time intervals: 1, 2, 5, 15, 30, 60 minutes shows the presence of orange coloured BAPI crystals precipitated on the bottom of the vial after a day.

10 Role of water during the conversion of PbO_2 to BAPI

The role of water during the formation of perovskites has been a controversial topic.[2] Prior works show both detrimental and beneficial effects of water on 2D perovskites, such as degradation[3,4], formation of pinholes in spincoated films[5] and defect passivation and stable device performance.[6] These different effects are usually attributed to water (either

atmospheric or controlled addition to the precursor solution) that is interacting during the formation of the perovskites. In this work, we perform the conversion experiments of both PbO_2 and PbI_2 under dry N_2 atmosphere, where water only appears as a by-product during the in-situ reduction of PbO_2. We believe that small quantities of water have a catalytic effect on the BAPI formation, as water reacts with BAI precursor to make more iodide species.

$$2\,H_2O + 2\,[C_4H_9NH_3I] \longrightarrow {}_2H_3O^+ + 2\,I^- + C_4H_9NH_2$$

These additional iodide species further assist in the formation of highly-valent lead iodide complexes, thus promoting the formation of BAPI microplates. To fully proof this more investigation is needed, which is outside the scope of this work.

References

1 G. Grimaldi, L. S. D. Antony, L. Helmbrecht, A. van der Weijden, S. W. van Dongen, I. Schuringa, J. Borchert, E. Alarcón-Lladó, W. L. Noorduin and B. Ehrler, *Applied Physics Letters*, 2021, **119**, 223102.

2 S. Xiao, K. Zhang, S. Zheng and S. Yang, 2020, **5**, 1147.

3 S. Wozny, M. Yang, A. M. Nardes, C. C. Mercado, S. Ferrere, M. O. Reese, W. Zhou and K. Zhu, 2015.

4 Y. H. Kye, C. J. Yu, U. G. Jong, Y. Chen and A. Walsh, *Journal of Physical Chemistry Letters*, 2018, **9**, 2196–2201.

5 H. Gao, C. Bao, F. Li, T. Yu, J. Yang, W. Zhu, X. Zhou, G. Fu and Z. Zou, 2015.

6 B. Conings, A. Babayigit, T. Vangerven, J. D'Haen, J. Manca and H.-G. Boyen, *Journal of Materials Chemistry A*, 2015, **3**, 19123–19128.
